# Effects of partially replacing dietary corn with sugars in a dual-flow continuous culture system on the ruminal microbiome

**DOI:** 10.1093/tas/txad011

**Published:** 2023-01-24

**Authors:** Anay D Ravelo, Jose A Arce-Cordero, Richard R Lobo, Ting Liu, Kwang C Jeong, Antonio Faciola

**Affiliations:** Department of Veterinary Population Medicine, College of Veterinary Medicine, University of Minnesota, St Paul, MN 55108, USA; Department of Animal Sciences, University of Florida, Gainesville, FL, 32611, USA; Department of Animal Sciences, University of Florida, Gainesville, FL, 32611, USA; Escuela de Zootecnia, Universidad de Costa Rica, San Jose, 11501-2060, Costa Rica; Department of Animal Sciences, University of Florida, Gainesville, FL, 32611, USA; Department of Animal Sciences, University of Florida, Gainesville, FL, 32611, USA; Emerging Pathogens Institute, University of Florida, Gainesville, FL 32611, USA; Department of Animal Sciences, University of Florida, Gainesville, FL, 32611, USA; Emerging Pathogens Institute, University of Florida, Gainesville, FL 32611, USA; Department of Animal Sciences, University of Florida, Gainesville, FL, 32611, USA

**Keywords:** 16S rRNA, starch, sugar, treated whey

## Abstract

The objective of this study was to evaluate the effects of feeding sugars as a replacement for starch on the ruminal microbiome using a dual-flow continuous culture system. Four periods of 10 days each were conducted with 8 fermenters in a 4 × 4 replicated Latin square design. Treatments included: 1) control with corn—CON, 2) molasses—MOL, 3) untreated condensed whey permeate—CWP, and 4) CWP treated with a caustic agent—TCWP as a partial substitute for corn. Sugars were defined as the water-soluble carbohydrates (WSC) concentration. Diets were formulated by replacing 4% of the diet DM in the form of starch from corn with the sugars in byproducts. Microbial samples for DNA analysis were collected from the solid and liquid effluent containers at 3, 6, and 9 h after feeding. Bacterial community composition was analyzed with sequencing the V4 region of the 16S rRNA gene using Illumina MiSeq platform. Data were analyzed with R 4.1.3 packages vegan, lmer, and ggplot to determine the effects of treatment on the relative abundance of taxa in the solid and liquid fractions, as well as the correlation of Acetate: Propionate ratio and pH to taxa relative abundance. Treatments did not affect alpha or beta diversity. At the phylum level the relative abundance of *Proteobacteria* was increased in CON compared to sugars in the solid fraction. In the liquid fraction, *Firmicutes* had greater relative abundance in sugar treatments while *Bacteroidota* and *Spirochaetota* were present in lower relative abundance in CWP. For solid and liquid samples, the family *Lachnospiraceae* had greater relative abundance in sugar treatments compared to CON. The decreased relative abundance of *Christensenellaceae* and *Rikenellaceae* paired with the greater relative abundance of *Selenomonadaceae* in CWP could help explain greater propionate molar proportion and decreased ruminal pH previously observed for this treatment. The genera *Olsenella* a lactic acid-producing bacterium, had the greatest relative abundance in MOL. Incorporating TCWP or MOL as a partial replacement for starch was more conservative of fibrolytic bacterial taxa compared to CWP. Additionally, TCWP did not increase bacterial taxa associated with synthesis of lactate as compared to MOL. Overall, replacing starch with sugars is mostly conservative of the ruminal microbiome; however, changes observed coincide with differences observed in acetate and propionate proportions and ruminal pH.

## INTRODUCTION

Corn grain is a feedstuff that is commonly fed to dairy cattle and up to 70% of the DM in the corn grain can be starch (Ferraretto et al., 2013). Starch can be included at 20–30% of the DM of a dairy cow diet (Ishler and Becker, 2021) as a source of energy, and can increase ruminal propionate. However, the inclusion of grain in the diets at high concentrations may lead to acidosis (Stone, 2004). Studies have shown that feeding high starch diets can alter the ruminal microbiome in favor of amylolytic and lactic acid-producing populations at the expense of fibrolytic bacteria (McCann et al., 2016). This shift in the ruminal microbiome can lead to reductions in ruminal pH, NDF digestibility and feed efficiency. Alternative sources of energy that could be potential replacements for starch in the diet include sugar byproducts such as molasses and whey (Oba, 2011; Ravelo et al., 2022).

Molasses is a byproduct of sugar production and is composed mainly of the sugar sucrose (Nikodinovic-Runic et al., 2013). Whey is a byproduct of cheese production, and it is composed mainly of lactose (Cassano et al., 2019). Whey permeate is a product produced by the removal of proteins and other solids from whey through ultrafiltration. Both molasses and whey permeate can be added as a partial replacement of corn grain in dairy cattle diets while maintaining nutrient digestibility (Penner and Oba, 2009). Sugars can be added in the diet from 1.5 to 8% of the DM of the lactating dairy cow diet (de Ondarza et al., 2017). In our companion study, condensed whey permeate, treated with a caustic agent, allowed for a similar ruminal microbial fermentation to molasses (Ravelo et al., 2021).

Although sugars contain highly fermentable carbohydrates that can be fermented faster than starch and can potentially increase the production of VFA and decrease ruminal pH, detrimental effects to ruminal pH have not been previously observed when starch is replaced with sugars (DeFrain et al., 2004; Broderick et al., 2008). It is unknown if these beneficial effects in ruminal fermentation when sugars are fed are related to modifications in the ruminal microbiome; therefore, we sought to elucidate this gap in knowledge. Therefore, the objective of this study was to evaluate the effects of partially replacing corn grain with molasses and condensed whey permeate on the ruminal microbiome using a dual-flow continuous culture system. Furthermore, we aimed to evaluate if treating condensed whey permeate with a caustic agent affects the ruminal microbiome. We hypothesized that partial replacement of the starch from corn with sugars from byproducts can help maintain bacterial populations in the ruminal microbiome that are associated with acetate, fiber fermentation, and ruminal pH.

## MATERIALS AND METHODS

The University of Florida Institutional Animal Use and Care Committee approved all the procedures for animal care and handling required for this experiment.

### Experimental Design and Diets

A detailed description of the experimental design is provided in our companion study (Ravelo et at., 2021). Briefly, the design consisted of four fermentation periods of 10 d each consisting of 7 d of adaption and 3 d of sampling. Eight dual-flow continuous culture fermenters were used in a replicated 4 × 4 Latin square design. Four experimental diets with differing sources of carbohydrates (Table 1) were randomized and fed to each fermenter. Experimental diets were formulated according to the NRC (2001) recommendation for a lactating Holstein cow with 680 kg body weight, producing 45 kg of milk per day with a milk fat, protein, and lactose percentage of 3.5%, 3.0%, and 4.8%, respectively. Additionally, diets were formulated for similar nutrient composition, only differing in the source of carbohydrate supplied as either a portion of starch or water-soluble carbohydrates (WSC). The control diet (CON) was formulated with ground corn grain, while the other three diets were formulated by replacing ground corn grain with either sugarcane molasses (MOL), condensed whey permeate (CWP), or treated condensed whey permeate (TCWP). A replacement factor of 4% was used to partially replace starch from corn for sugars from the byproducts, this allowed the inclusion rate of the byproducts to be within 1.5–8% DM (de Ondarza et al., 2017), an acceptable rate for inclusion of sugars in diets for dairy cows. Whey permeate was treated with 0.5% by weight of sodium hydroxide to 32% by weight of lactose to raise the pH to 8, stabilizing the crystallization of lactose, for improving its shelf life (US Patent number 8,182,848 B2; Harris and Mostyn, 2012).

**Table 1.** Ingredient and chemical composition of the experimental diets

Item, %DM	Diets^1^			
	CON	MOL	CWP	TCWP
Ingredient composition				
Corn silage	41.7	41.7	41.7	41.7
Alfalfa hay	18.0	18.0	18.2	18.2
Soybean meal	8.0	8.0	8.0	8.0
Canola meal	8.0	8.0	8.0	8.0
Corn grain	22.0	15.4	17.4	17.2
Molasses	–	6.58	–	–
Untreated condensed whey permeate	–	–	4.37	–
Treated condensed whey permeate	–	–	–	4.61
Mineral premix	1.45	1.45	1.45	1.45
Calcium carbonate	0.55	0.55	0.55	0.55
Urea	0.32	0.32	0.32	0.32
Chemical composition^3^				
OM	92.8	92.3	92.5	92.4
CP	17.1	16.8	16.9	16.9
RDP^2^	10.9	10.8	10.9	10.9
RUP^2^	6.20	6.00	6.00	6.00
NDF	28.7	28.2	28.4	28.4
ADF	17.5	17.3	17.5	17.5
NFC^4^	44.8	45.1	44.9	44.9
NSC^5^	35.2	35.1	35.9	35.8
Starch	30.9	26.9	27.7	27.6
WSC^6^	4.30	8.17	8.22	8.23
EE	2.33	2.21	2.25	2.23
NE_L_, Mcal/kg DM^2^	1.59	1.58	1.59	1.52

^1^CON, control; MOL, molasses; CWP, untreated condensed whey permeate; TCWP, treated condensed whey permeate.

^2^Estimated using the NRC (2001) model.

^3^Expressed as %DM unless otherwise stated.

^4^NFC, non-fibrous carbohydrates.

^5^NSC, non-structural carbohydrates = NFC + WSC.

^6^WSC, water-soluble carbohydrates (Deraiz 1961).

Samples for feed ingredients were ground through a 1 mm screen and sent to Rock River Laboratories (Watertown, WI, 53094) for nutrient composition analysis. Samples were analyzed for DM (Shreve et al., 2006); ash (AOAC, 1990; method 942.05); NDF and ADF were analyzed sequentially (Van Soest et al., 1991) with heat-stable α-amylase and sodium sulfite for NDF; starch (Hall, 2008); WSC (Dubois et al., 1956); crude fat (AOAC, 2017; Modified AOCS Am5-04); and CP (AOAC, 2000; method 990.03). The liquid feed samples and corn chemical composition is included in Table 2. The chemical composition of individual feed ingredients was used to formulate the experimental diets. Corn silage was dried for 72 h in a 60 °C forced-air oven, then ground to 2 mm along with soybean meal and corn grain to be used in the diets. Each fermenter was fed the corresponding diet of 106 g of DM/d divided in two feeding times, at 0800 and 1800. Liquid feeds (molasses and whey permeate) were measured and dosed into each corresponding fermenter right before the dry ingredients were fed.

**Table 2.** Partial chemical composition of corn grain versus sugar containing byproducts used in the experimental diets testing the substitution of starch with sugars in a dual-flow continuous culture system

Chemical composition^1^	Ingredient			
	Corn	Molasses	CWP^2^	TCWP^3^
DM	92.9	69.8	32.3	29.5
CP	8.09	4.03	3.10	3.34
Starch	68.1	7.48	ND^4^	0.79
WSC^4^	2.07	60.8	91.7	86.9
EE	2.20	0.29	0.31	ND^5^
Ash	1.51	10.6	9.35	10.1

^1^Expressed as %DM unless otherwise stated.

^2^CWP, condensed whey permeate.

^3^TCWP, condensed whey permeate treated with a caustic agent.

^4^WSC, water-soluble carbohydrates (Deraiz 1961).

^5^ND, not detected.

### Dual-flow Continuous Culture System Operation

A dual-flow continuous culture system as developed by Hoover et al. (1976) and described by Arce-Cordero et al. (2021a, 2021b) was used for this experiment. Artificial saliva was infused continuously (Weller and Pilgrim, 1974) at a rate of 3.1 mL/min into the system and a partial removal of fermentation liquid effluent at 1.55 mL/min, allowed the removal of liquid and solid effluents to be controlled at a rate of 11% and 5.5%/h respectively. An anaerobic environment (200 mL of N_2_/min) was maintained with constant infusion of N_2_ gas, and constant temperature (39 °C) and agitation (100 rpm) were controlled throughout.

Ruminal contents were collected from two ruminally cannulated Holstein cows consuming a similar diet (DM basis: 60% whole plant corn silage, 12.5% ground corn, 13% citrus pulp, 12% soybean meal, and 2.5% mineral and vitamin mix) to the control diet. Contents were collected from both cows approximately 1 h after feeding and filtered through four layers of cheesecloth into prewarmed thermos flasks for transportation to the lab. Each fermenter was prewarmed and had infusion of N_2_ gas before inoculation. A ratio of 50:50 ruminal content from the cows was used to inoculate each fermenter. On d 5 of each period, artificial saliva was switched for ^15^N enriched saliva with 1.54 g of ammonium sulfate N^15^/20 L to replace 0.71 g of urea. To create a steady state of ^15^N prior to changing the saliva, a pulse dose of labeled ammonium sulfate provided 0.1733 g of 10.2% excess (^15^NH_4_)_2_SO_4_ (Sigma-Aldrich Co, St. Louis, MO, 63103) per fermenter. Ammonium sulfate was continuously added to the system as a marker in the artificial saliva at a rate of 0.077 g/L until the end of each experimental period. To prevent further microbial fermentation the solid and liquid effluent containers were placed in an ice bath at 1 °C on d 7. At the end of d 10, the fermenters were disassembled, cleaned, and reassembled for the following period.

### Collection of Samples

The first 7 days of each period were used for adaptation and stabilization of the bacterial communities. Collection of samples was conducted on the last 3 days of the period which consisted of days 8, 9, and 10 of fermentation. The pH of each fermenter was measured throughout each sampling day at 0, 1, 2, 4, 6, and 8 h after morning feeding.

Bacterial samples were collected from the individual liquid and solid effluent containers at 3, 6, and 9, h after feeding on d 8, 9, and 10. A 15 mL sample from the liquid effluent was collected at each time point to make a composite sample of the time points per day of the liquid associated bacteria (LAB) collected from the filtered liquid effluent totaling 45 mL per day. The contents from the solid effluent containers were strained through four layers of cheese cloth and from the remaining solid particles approximately 25 g were collected for the particulate associate bacteria (PAB) and composited throughout each time point in the day. All samples were stored in −80 °C for DNA extraction and sequencing.

At the end of each sampling day the effluent container contents were combined and mixed for 30 s using a handheld mixer. An aliquot of the mixture was strained through four layers of cheese cloth to obtain a 10 mL sample acidified with 0.1 mL of 50% sulfuric acid. The samples were all stored at −20 °C for subsequent VFA analysis.

### Acetate and Propionate Concentration

The samples for VFA were processed following the Ruiz-Moreno et al. (2015) method. The samples were centrifuged at 10,000 × *g* for 15 min at 4 °C, and the supernatant was collected for analysis. A crotonic and metaphosphoric acid solution was added to the supernatant at a 1:5 ratio and then allowed to freeze overnight. After thawing the samples were centrifuged at 10,000 × *g* for 15 min at 4 °C. Ethyl acetate was mixed into the supernatant in a 2:1 ratio, vortexed, and allowed to settle. The top layer was transferred to a chromatography vial and analyzed using gas chromatography (Agilent 7820A GC, Agilent Technologies, Palo Alto, CA) with a flame ionization detector and a capillary column (CP-WA × 58 FFAP 25 m 0.53 mm, Varian CP7767, Varian Analytical Instruments, Walnut Creek, CA). The column was maintained at 110 °C with the injector temperature at 200 °C and the detector at 220 °C.

### DNA Extraction

For the DNA extraction, the DNA samples were thawed and were composited by time points within days to be extracted using a microbial DNA extraction kit (D6010 Quick-DNA Fecal/Soil microbe DNA MiniPrep Kit, Zymo Research Corporation, Irvine, CA). A total of 32 composite solid samples and 32 composite liquid samples were processed for analysis. Briefly, about 200 mg or mL of each sample was placed into a lysis tube with a buffer and bashing beads. The lysis tube was processed through a beat beater (Bead Mill Homogenizer, Bertin, France). Then the supernatant was recovered and processed through several centrifugation and filtration steps until the final eluted DNA was filtered into a clean microcentrifuge tube. Before storage of extracted samples in −80 °C, a Qubit^®^ Fluorometer (Invitrogen, San Diego, CA. USA) was used to measure the DNA concentration in each sample (Arce-Cordero et al., 2022a, 2022b).

### DNA Amplification and Sequencing

Procedures for DNA amplification of the V4 variable region of the bacterial 16S rRNA gene using dual-index primers (Caporaso et al., 2011) were performed in accordance with Kozich et al. (2013). A polymerase chain reaction (PCR) was performed consisting of 1 µL forward index primer, 1 µL reverse index primer, 1 µL DNA template (10 ng/µL), and 17 µL Pfx AccuPrime master mix (Invitrogen, San Diego, CA, USA) in a Bio Rad C1000 Touch^TM^ Thermal cycler (BIO-RAD, Hercules, CA, USA) to amplify the DNA. The following procedure was followed denaturation of 5 min at 95 °C, followed by 30 cycles of 30 s at 95 °C, 30 s at 55 °C for annealing, 1 min at 72 °C for extension and 5 min for elongation at 72 °C. Contaminants such as small DNA fragment and primers were removed using a 1% low melting agarose gel extraction kit (National Diagnostics, Atlanta, GA, USA). Amplicons were purified and normalized with a SequalPrep Normalization Plate Kit (Applied Biosystems, Foster City, CA) for the construction of the pooled DNA library, and the DNA concentration was read with a Qubit^®^ Fluorometer (Invitrogen, San Diego, CA, USA). There were 32 samples from the particulate phase and 32 from the liquid phase to be sequenced. Adapters were added to the amplicons, and the DNA library was constructed by pooling equally the amplicons together and using quantitative real-time PCR for the final quality check evaluation. Sequencing was performed using a MiSeq reagent kit V2 (2 × 250 cycles run; Illumina, San Diego, CA, USA) in a Illumina MiSeq platform (Illumina, San Diego, CA, USA) by the Interdisciplinary Center for Biotechnology Research (ICBR) of the University of Florida. The amplicon sequencing data for the 16S rRNA gene were deposited into the NCBI database (accession number PRJNA854650).

### Bacterial Sequence Data Analysis

The sequencing data were analyzed with Quantitative Insights into Microbial Ecology version 2 (QIIME 2) pipeline (Bolyen et al., 2019). The forward and reverse raw reads for each sample was imported and the quality of the bases was evaluated using the Interactive Quality Plot. The Divisive Amplicon Denoising Algorithm (DADA2) pipeline was implemented in the QIIME 2. It was used for sequence quality control which included steps to filter low quality reads, denoise reads, merge the paired-end reads, and remove chimeric reads from the sequence. The align-to-tree-mafft-fasttree pipeline from the q2-phylogeny plugin of QIIME 2 was used to create a phylogenetic tree. Sequencing depth was normalized to 10,800 per sample and the number of amplicon sequence variants (ASVs), diversity (Shannon Index), richness (Chao1), and Bray-Curtis distance were calculated by the core metrics-phylogenetic method. The ASVs were classified into phylum, class, order, family, and genus using the SILVA 138 database (https://www.arb-silva.de/documentation/release-1381/). Average relative abundances greater than 0.1% in the samples were considered for the analysis.

### Statistical Analyses

Data were analyzed with R 4.1.3. The results of the Bray–Curtis distance were analyzed with the R package vegan (Callahan et al., 2016) and visualized with principal component analysis (PCoA). The effects of treatment on community structures were determined using the PERMANOVA test implemented in QIIME 2. Alpha diversity and log-transformed data of taxa relative abundance were analyzed using a mixed model with the lme4 package of R (Bates et al., 2015). The statical model included the fixed effect of treatment and the random effects of period, square, and fermenter within square. The correlations of the acetate: propionate (A:P) ratio and pH with the relative abundance of bacterial taxa affected by treatment were analyzed and visualized using Pearson correlation and ggplot2 (Wickham, 2016). Significance was declared at *P* ≤ 0.05, while 0.05 < *P* ≤ 0.10 were considered trends.

## RESULTS AND DISCUSSION

Detailed ruminal fermentation results, nutrient digestibility, and N partitioning data are included in our companion study (Ravelo et at., 2021). For this study, a total of 64 samples, 32 solid phase samples and 32 liquid phase samples, were sequenced. Due to a low number of sequencing reads (315) from one solid CON sample, a total of 31 solid samples and 32 liquid samples were used for analysis. After the process of filtering, denoising, merging, and removing with the DADA2 pipeline a total of 1,318,665 high-quality sequences were preserved. For the solid samples there were 20 phyla, 33 classes, 61 orders, 94 families, and 223 genera detected. For the liquid samples there were 19 phyla, 33 classes, 69 orders, 96 families, and 207 genera detected.

**Figure 1.**
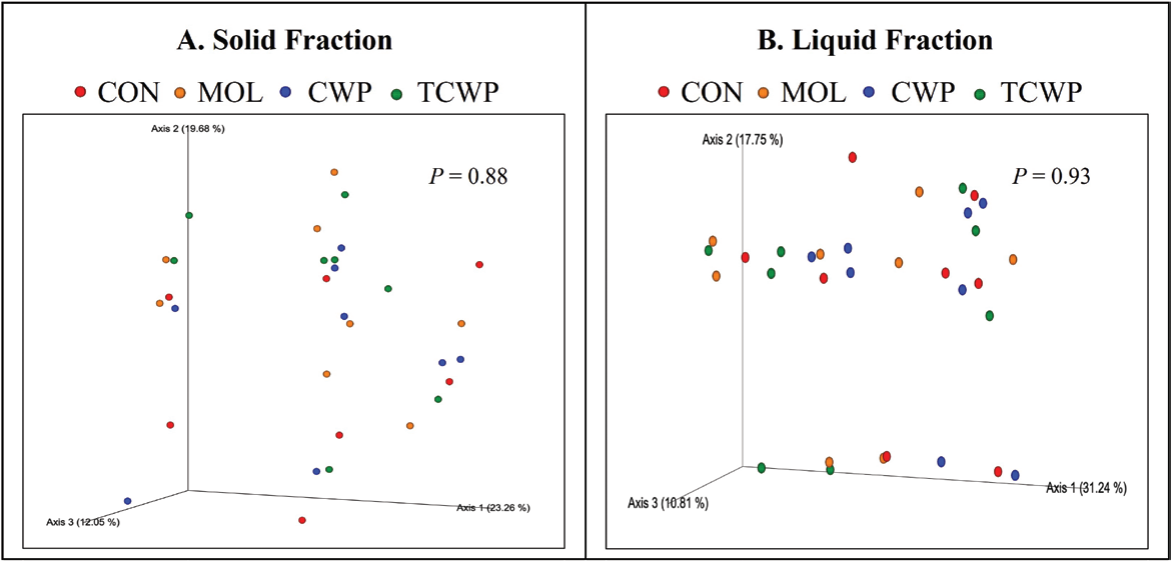
Principal coordinates analysis (PCoA) plots of the Bray–Curtis similarity that compares the effect of treatment on the ruminal bacterial community structures. Treatments: CON-was formulated with corn (starch); MOL-was formulated with molasses partially replacing 4% of diet DM of starch from corn with sugar from molasses; CWP-was formulated with condensed whey permeate partially replacing 4% of diet DM of starch from corn with sugar from CWP; TCWP-was formulated with treated CWP partially replacing 4% of diet DM of starch from corn with sugar from CWP treated with a caustic agent.

**Figure 2.**
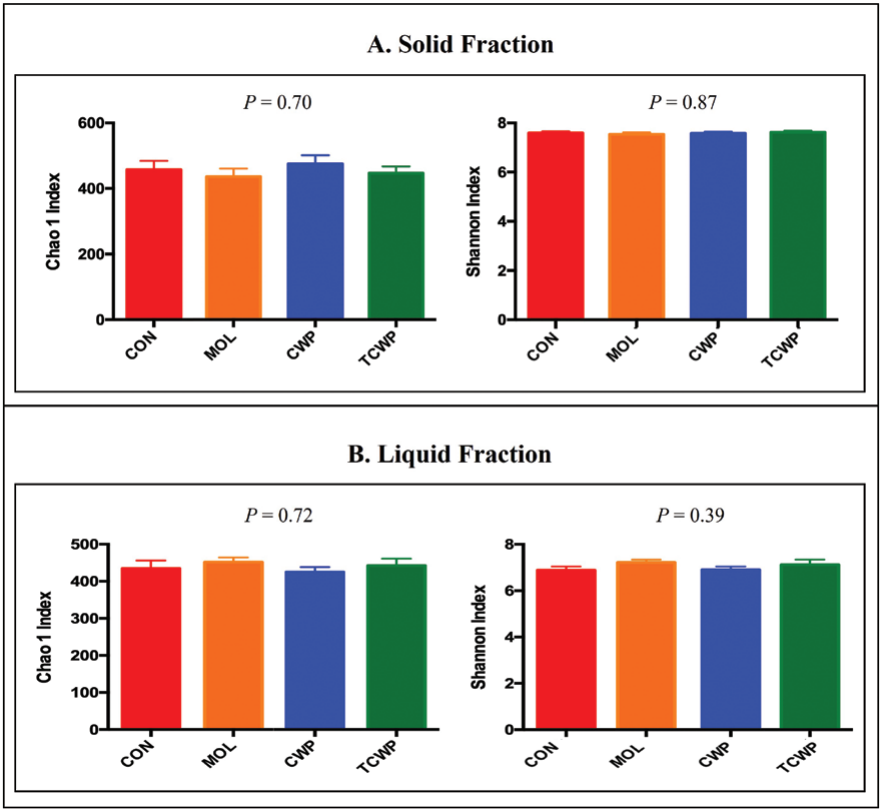
The alpha diversity effects of each treatment represented as Chao1 and Shannon Index. Treatments: CON-was formulated with corn (starch); MOL-was formulated with molasses partially replacing 4% of diet DM of starch from corn with sugar from molasses; CWP-was formulated with CWP partially replacing 4% of diet DM of starch from corn with sugar from CWP; TCWP-was formulated with treated CWP partially replacing 4% of diet DM of starch from corn with sugar from CWP treated with a caustic agent.

The bacterial communities of each treatment using Bray–Curtis similarity index are presented in Figure 1. According to the index there was no effect of treatment on both the solid and liquid fraction which means there are no differences in bacterial populations between the treatments. Likewise, for the alpha diversity depicted in Figure 2, treatments had no effect on the Chao1 or Shannon indices, indicating that the replacement of starch by sugars did not have an effect on the richness or evenness of the microbiome. The relative abundance of bacteria at each taxonomic level was considered to help understand the possible effects of replacing starch for sugar sources on the ruminal microbiome.

In Table 3, the phyla relative abundances for the solid and liquid samples are depicted. Overall, the relative abundances of solid and liquid phyla presented in this study are similar to those reported in other studies that considered the relative abundances present in the solid and liquid phases of ruminal fluid in a dual-flow continuous culture system (Arce-Cordero et al., 2022). For the solid fraction there were six major phyla detected: *Firmicutes*, *Bacteroidota*, *Spirochaetota*, *Proteobacteria*, *Actinobacteriota*, and *Fibrobacterota*. There was an effect of treatment on the relative abundance of *Proteobacteria* in the solid samples where the relative abundance of this phylum was decreased in the MOL (*P* = 0.06) and TCWP (*P* = 0.03) compared to CON. *Proteobacteria* has been associated with dysbiosis of the gut microbiome (Shin et al., 2015). Similarly, a high ratio of *Proteobacteria* to the collective *Firmicutes* and *Bacteroidetes* is also indicator of rumen dysbiosis and this unbalance of microbial community can be associated with lower ruminal pH (Auffret et al., 2017). These changes correspond with the pH differences observed in the treatments in our companion paper (Ravelo et al., 2021). The treatments MOL and TCWP with the decreased relative abundance of *Proteobacteria* were also the ones with greater ruminal pH, which suggests that the inclusion of sugars may help maintain rumen eubiosis.

**Table 3.** Effects of replacement of corn grain with sugar containing byproducts on relative abundance of main phylum of bacteria in solid and liquid fractions

Phylum	Treatment Means^1^				SEM	*P*-value^2^
	CON	MOL	CWP	TCWP		
Solid fraction						
Firmicutes	54.4	56.9	55.5	57.1	1.64	0.26
Bacteroidota	21.6	19.7	20.8	20.4	1.38	0.51
Spirochaetota	11.0	11.9	11.2	10.6	1.07	0.58
Proteobacteria	5.89^a^	3.54^b^	4.83^a,b^	3.29^b^	1.21	0.02
Actinobacteriota	1.96	3.00	3.15	3.01	0.68	0.23
Fibrobacterota	2.23	2.14	1.90	2.17	0.25	0.78
Liquid fraction						
Firmicutes	37.0	40.8	40.5	40.3	2.13	0.07
Bacteroidota	28.8	28.4	26.7	29.9	1.85	0.10
Proteobacteria	23.2	18.1	22.1	18.3	3.26	0.15
Spirochaetota	6.28^a,b^	7.33^a^	4.99^b^	5.87^a,b^	1.36	0.03
Actinobacteriota	2.03	2.62	3.43	2.76	0.69	0.19

^1^CON, control; MOL, molasses; CWP, untreated condensed whey permeate; TCWP, treated condensed whey permeate.

^2^Effect of experimental treatment.

^a-b^Means within a row with different subscripts differ (P ≤ 0.05).

For the liquid fraction there were five major phyla detected: *Firmicutes*, *Bacteroidota*, *Proteobacteria*, *Spirochaetota*, and *Actinobacteriota*. The relative abundance of *Firmicutes,* which can be butyrate-producing bacteria (Louis and Flint, 2009), tended to be greater in the sugar diets (*P* = 0.07). With the inclusion of sugar in dairy cow diets some studies have seen an increase in butyrate concentration (Chamberlain et al., 1993; de Ondarza et al., 2017); however, this was not an observed affect in our companion study (Ravelo et al., 2021) where butyrate molar proportion was not affected by sugar inclusion. There was a tendency in *Bacteroidota* (*P* = 0.10) where CWP had the lowest relative abundance observed. Some bacteria in the phylum *Bacteroidota* have been associated to hemicellulose break down (Naas et al., 2014; Hernández et al., 2022) in ruminal fluid. This agrees with CWP having the lowest observed ruminal pH. Low ruminal pH, below 6.0, has been associated with decreased fiber degradation (Dijkstra et al., 2012) in ruminal fluid. The inclusion of CWP in the diet could potentially decrease fiber digestion. However, when CWP is treated the decrease in ruminal pH was not observed, thus treatment could be conservative to fiber digestibility. Additionally, there was a difference in the relative abundance of *Spirochaetota* where there was more present in MOL compared to CWP (*P* = 0.02). Bacteria in the *Spirochaetota* phylum in the rumen of cattle have been characterized as pectin and hemicellulose degrading (Gharechahi et al., 2021). Thus, molasses may be a better sugar alternative for starch compared to condensed whey since it may help maintain fiber degradation.

The main families in the solid and liquid fractions are presented in Table 4. In both the solid (*P* < 0.01) and liquid (*P* < 0.01) fractions, the relative abundance of *Lachnospiraceae* was increased in the treatments that supplemented sugars compared to CON. *Lachnospiraceae* family are bacteria that have been associated with the fermentation of pectin (Cotta and Forster, 2006) and is a butyrate-producing *Firmicutes* (Bach et al., 2019). In the solid samples, the relative abundance of *Succinivibrionaceae* (*P* = 0.02) and *Clostridia_UCG-014* (*P* = 0.02) were greater in CON compared to both MOL and TCWP. The *Succinivibrionaceae* family has been positively correlated with propionate (Xue et al., 2019) which concords with the results observed in our companion study (Ravelo et al., 2021) where the propionate molar proportion was greater in CON compared to MOL and TCWP. Similarly, *Clostridia_UCG-014* has also been positively correlated with propionate in mice fecal samples (He et al., 2022). Additionally, the family *Christensenellaceae* tended to be decreased in CWP, this family has been associated with the fermentation of structural carbohydrates producing acetate and butyrate as end products (Morotomi et al., 2012). Concordantly, CWP had the lowest numerical molar proportion of butyrate and ruminal pH, thus condensed whey could potentially hinder fiber degradation.

**Table 4.** Effects of replacement of corn grain with sugar containing byproducts on relative abundance of main families of bacteria in solid and liquid fractions

Family	Treatment Means^1^				SEM	*P*-value^2^
	CON	MOL	CWP	TCWP		
Solid						
Lachnospiraceae	29.9^b^	34.6^a^	32.9^a^	33.8^a^	1.11	< 0.01
Prevotellaceae	18.0	15.7	17.5	16.2	1.33	0.20
Spirochaetaceae	11.1	11.8	11.1	10.7	1.08	0.65
Succinivibrionaceae	5.47^a^	3.07^b^	4.60^a,b^	2.87^b^	1.24	0.02
Acidaminococcaceae	4.50	4.56	4.28	4.69	0.47	0.33
Selenomonadaceae	3.88	3.60	3.74	3.83	0.37	0.87
Ruminococcaceae	2.75	2.37	2.50	2.09	0.27	0.15
Fibrobacteraceae	2.24	2.15	1.88	2.20	0.25	0.71
Christensenellaceae	2.04	2.10	1.76	2.40	0.21	0.07
Clostridia_UCG-014	1.96^a^	1.12^b^	1.54^a,b^	1.14^b^	0.31	0.02
Liquid						
Prevotellaceae	22.8	20.8	21.6	22.4	1.40	0.09
Succinivibrionaceae	22.7	17.1	21.6	17.6	3.26	0.13
Lachnospiraceae	14.7^b^	19.0^a^	18.8^a^	18.6^a^	1.13	< 0.01
Spirochaetaceae	6.27^a,b^	7.25^a^	4.95^b^	5.85^a,b^	1.35	0.02
Selenomonadaceae	5.14^a,b^	4.19^a,b^	5.94^a^	3.92^b^	0.83	0.05
Rikenellaceae	2.33^a,b^	2.81^a,b^	2.09^b^	2.89^a^	0.34	0.05
Oscillospiraceae	2.45^a,b^	2.74^a^	1.82^b^	2.87^a^	0.52	< 0.01
Acidaminococcaceae	2.65	2.70	2.57	2.81	0.27	0.75
Bifidobacteriaceae	1.50	1.63	2.78	2.02	0.63	0.18
Ruminococcaceae	1.75	1.70	1.53	1.56	0.34	0.86

^1^CON, control; MOL, molasses; CWP, untreated condensed whey permeate; TCWP, treated condensed whey permeate.

^2^Effect of experimental treatment.

^a-b^Means within a row with different subscripts differ (P ≤ 0.05).

For the liquid fraction samples, *Prevotellaceae* family relative abundance tended to be depressed in MOL, this family is known for enzymes involved in propionate and acetate formation in the ruminal microbiome (Deusch et al., 2017). Less production of acetate and propionate might be partially responsible for the lesser total VFA concentration observed for MOL in our companion paper (Ravelo et al., 2021). Additionally, the *Spirochaetaceae* family relative abundance was greater in MOL compared to CWP (*P* = 0.02), and some members of this family have LPS in their outer membrane as a principal surface antigen (Karami et al., 2014). For the *Selenomonadaceae* family, the relative abundance was less in TCWP compared to CWP (*P* = 0.05). This family has been negatively correlated with pH and positively correlated with propionate (Savin et al., 2022), which agrees with our companion study (Ravelo et al., 2021) where CWP had the lowest ruminal pH and had numerical greater propionate molar proportion compared to TCWP.

Similarly, members of the *Rikenellaceae* family have also been positively correlated with ruminal pH and acetate to propionate ratio (Qiu et al., 2022). Their relative abundance was greater in TCWP compared to CWP (*P* = 0.07) and TCWP had both greater ruminal pH and acetate to propionate ration compared to CWP. Finally, the relative abundance of *Oscillospiraceae* was greater in TCWP and MOL compared to CWP (*P* < 0.01; *P* = 0.02). This family has been positively correlated with NDF levels in the diet in the rumen (Wei et al., 2021) and cellulolytic bacteria. It is possible that treated CWP and molasses are sugars that are more protective for fiber digestibility than CWP.

The genera affected by treatments in the solid fraction are depicted in Table 5. There was a total of 15 genera that had significant differences due to treatment, 7 are from the family *Lachnospiraceae,* 2 from *Ruminococcaceae,* and 1 each from *Prevotellaceae*, *Christensenellaceae*, *Succinivibrionaceae*, *Clostridia_UCG-014*, *Selenomonadaceae,* and *Atopobiaceae*. This closely follows the differences observed in the families for the solid fraction. For the genus *Acetitomaculum,* the relative abundance of this acetogen was increased in the sugar diets compared to CON (*P* < 0.01). In the past, using reductive acetogenesis in the rumen was investigated as a way to decrease methane emissions as a potential hydrogen sink; however, acetogenesis has been considered to be less favorable than propionate (Lopez et al., 1999). Potentially the increase in this genus might indicate less methane emission for the diets that include sugar, but this was not measured in this study.

**Table 5.** Effects of replacement of corn grain with sugar containing byproducts on relative abundance of main genera of bacteria in solid fractions

Genus	Family	Treatment means^1^				SEM	*P*-value^2^
		CON	MOL	CWP	TCWP		
*Prevotella*	Prevotellaceae	13.8	11.0	12.9	11.8	1.09	0.08
*Acetitomaculum*	Lachnospiraceae	5.05^b^	8.63^a^	8.99^a^	7.98^a^	0.86	< 0.01
*Lachnospiraceae NK3A20 group*	Lachnospiraceae	5.33^b^	6.19^a,b^	5.68^b^	6.80^a^	0.56	0.01
*[Eubacterium] ruminantium group*	Lachnospiraceae	1.13^a^	0.77^b^	1.11^a^	0.93^a,b^	0.14	0.01
*Lachnospiraceae NK4A136 group*	Lachnospiraceae	0.62^a,b^	0.75^a^	0.51^b^	0.61^a,b^	0.07	0.01
*Lachnospiraceae AC2044 group*	Lachnospiraceae	0.59^a,b^	0.63^a,b^	0.50^b^	0.76^a^	0.09	0.03
*Lachnospiraceae XPB1014 group*	Lachnospiraceae	0.38^a,b^	0.43^a,b^	0.29^b^	0.51^a^	0.09	0.01
*Syntrophococcus*	Lachnospiraceae	0.15^a,b^	0.08^b^	0.20^a^	0.12^a,b^	0.02	< 0.01
*Succinivibrionaceae UCG-001*	Succinivibrionaceae	4.31	1.84	3.71	1.66	1.23	0.06
*Christensenellaceae R-7 group*	Christensenellaceae	2.02	2.12	1.71	2.36	0.21	0.06
*Ruminococcus*	Ruminococcaceae	2.16^a^	1.71^a,b^	1.88^a,b^	1.51^b^	0.15	0.02
*CAG-352*	Ruminococcaceae	0.33^a,b^	0.12^b^	0.35^a^	0.34^a^	0.11	0.05
*Clostridia UCG-014*	Clostridia_UCG-014	1.96^a^	1.12^b^	1.58^a,b^	1.13^b^	0.31	0.02
*Veillonellaceae UCG-001*	Selenomonadaceae	0.77^b^	1.02^a^	0.78^b^	1.03^a^	0.11	0.02
*Olsenella*	Atopobiaceae	0.25^b^	0.47^a^	0.31^a,b^	0.27^b^	0.09	0.01

^1^CON, control; MOL, molasses; CWP, untreated condensed whey permeate; TCWP, treated condensed whey permeate.

^2^Effect of experimental treatment.

^a-b^Means within a row with different subscripts differ (P ≤ 0.05).

The genera *Lachnospiraceae NK3A20 group* and *Syntrophococcus* have been identified as core ruminal epithelial OTUs (Anderson et al., 2021), indicating that they may have a role in butyrate production. Similarly, *Lachnospiraceae NK4A136* group has been associated with butyric acid (Kheirandish et al., 2022). There was no clear pattern for genera from the *Lachnospiraceae* family, at least one genus had a greater relative abundance for one of each of the treatments compared to the others. This fluctuation in relative abundance may be partially responsible for the lack of treatment effects observed in butyrate molar proportion.

The relative abundance of the genus *Succinivibrionaceae_UCG-001* tended to be greater in CON and CWP compared to TCWP (*P* = 0.06). This genus has been identified to produce succinate a precursor for propionate (Holman and Gzyl, 2019). Similarly for the genus *Clostridia UCG-014,* the relative abundance was greater in CON compared to MOL and TCWP (*P* = 0.03; *P* = 0.04), as previous mentioned it has been positively correlated with propionate (He et al., 2022). These differences concur with results seen in our companion study (Ravelo et al., 2021) where propionate tended to be increased in CON.

The genus *Ruminococcus* has been reported to be present in the liquid and solid phase of the rumen (Kong et al., 2010) and some species in this genus are associated with fiber degradation. The relative abundance of this genus was greater in CON compared to TCWP (*P* = 0.02). However, there were no effect of fiber digestibility across treatments. Finally, a difference was observed in the genera *Olsenella,* there was greater relative abundance in MOL compared to CON and TCWP. *Osenella* has been shown to use glucose and produce lactic acid as an end product (Kraatz et al., 2011). This concords with results in our companion paper (Ravelo et al., 2021) where lactate concentration was greater in MOL compared to the other treatments. A Pearson correlation was conducted on A:P ratio and pH as the differences in relative abundance in solid genera seem to coincide with the changes observed in fermentation end products and ruminal pH.

**Figure 3.**
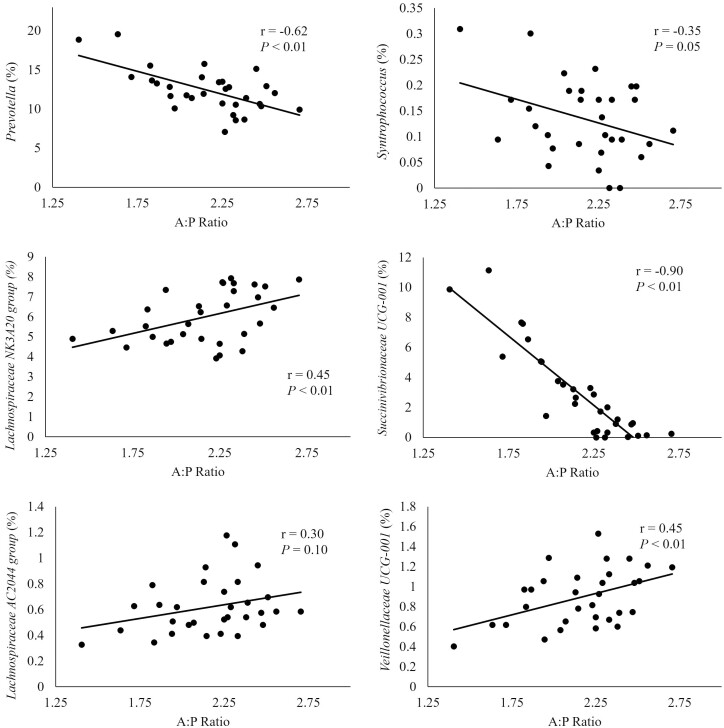
Correlation between acetate: propionate ratio (A:P ratio) in ruminal fluid and relative abundance of genera in the solid fraction. Moderate (> 0.30) to strong (> 0.50) correlations are represented.

The correlation of the A:P ratio with genera in the solid fraction, depicted in Figure 3, demonstrates that *Prevotella, Syntrophococcus,* and *Succinivibrionaceae_UCG-001,* were negatively correlated with A:P ratio (*r* = −0.62, −0.35, −0.90). This suggests that greater relative abundance of these bacteria promotes greater propionate production in comparison to acetate. The treatment that had increased relative abundances of these bacteria was CON, which corresponds to results observed in our companion study (Ravelo et al., 2021) were propionate molar proportion tended to be greater in CON. The genera *Lachnospiraceae NK3A20 group, Lachnospiraceae AC2044 group,* and *Veillonellaceae UCG-001* were positively correlated with A:P ratio (*r* = 0.45, 0.30, 0.45), suggesting that these bacteria can promote more acetate production compared to propionate. The relative abundance of these bacteria was increased in TCWP which corresponds to the tendency observed in our companion paper (Ravelo et al., 2021) were TCWP had greater molar proportion of acetate.

**Figure 4.**
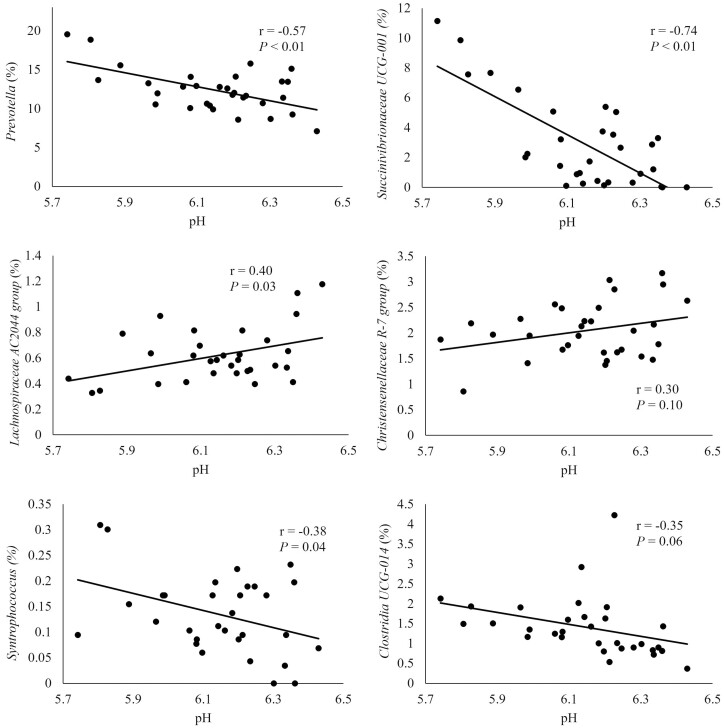
Correlation between pH in ruminal fluid and relative abundance of genera in the solid fraction. Moderate (> 0.30) to strong (> 0.50) correlations are represented.

The correlation of pH with genera in the solid fraction is depicted in Figure 4. The pH was negatively correlated with *Prevotella, Syntrophococcus, Succinivibrionaceae_UCG-001,* and *Clostridia UCG-014* (*r* = −0.57, −0.38, −0.74, −0.35). This suggests that in a higher pH the relative abundance of these bacteria was lower. Abundance of these bacteria was lower in MOL and TCWP which were the treatments observed to have greater pH. Likewise, the pH was positively correlated to *Lachnospiraceae AC2044 group* and *Christensenellaceae R-7 group* (*r* = 0.40, 0.30) which were in greater abundance in TCWP and MOL.

The genera affected by treatments in the liquid fraction are depicted in Table 6. Likewise, to the solid fraction it followed differences observed in the families. There were treatment effects on 16 genera, 5 belonging to *Lachnospiraceae,* 2 to *Prevotellaceae, Oscillospiraceae, and Selenomonadaceae,* and 1 each from *Succinivibrionaceae, Spirochaetaceae, Rikenellaceae*, *Ruminococcaceae,* and *Atopobiaceae.* The genus *Prevotella* has been characterized to utilize starch and proteins to produce acetate and succinate in the rumen (Xue et al., 2020). This genus had a relative abundance that was lower in MOL compared to CON (*P* = 0.02) and TCWP (*P* = 0.02). Although MOL had less starch than CON, its concentration of starch was similar to that in TCWP, hence the decrease in starch concentration in the diet when incorporating sugars alone cannot account for this difference. Thus, characteristics of treated condensed whey maybe more protective to *Prevotella* abundance compared to molasses; however, this requires further investigation. For *Prevotellaceae Ga6A1 group* the relative abundance was greater for MOL compared to CWP (*P* = 0.03) and TCWP (*P* = 0.02). This genus has been positively associated with feed efficiency in transition cows (Bach et al., 2019), thus molasses may be a more feed efficient sugar alternative for starch.

**Table 6.** Effects of replacement of corn grain with sugar containing byproducts on relative abundance of main genera of bacteria in liquid fractions

Genus	Family	Treatment Means^1^				SEM	*P*-value^2^
		CON	MOL	CWP	TCWP		
*Prevotella*	Prevotellaceae	18.6^a^	16.1^b^	18.1^ab^	18.6^a^	1.00	0.01
*Prevotellaceae Ga6A1 group*	Prevotellaceae	1.41^a,b^	1.70^a^	0.95^b^	0.92^b^	0.28	0.01
*Succinivibrionaceae UCG-001*	Succinivibrionaceae	17.0	9.13	17.1	9.49	3.73	0.06
*Treponema*	Spirochaetaceae	6.19^a,b^	7.14^a^	4.92^b^	5.74^a,b^	1.33	0.03
*Acetitomaculum*	Lachnospiraceae	2.54^b^	5.44^a^	5.55^a^	4.58^a^	0.46	< 0.01
*Lachnospiraceae NK3A20 group*	Lachnospiraceae	2.79^b^	4.03^a^	3.89^a^	4.72^a^	0.41	< 0.01
*Marvinbryantia*	Lachnospiraceae	0.18^a,b^	0.26^a^	0.14^b^	0.25^a^	0.05	0.02
*Lachnospiraceae AC2044 group*	Lachnospiraceae	0.15	0.21	0.15	0.22	0.04	0.07
*Oribacterium*	Lachnospiraceae	0.16^a^	0.09^b^	0.10^a,b^	0.11^a,b^	0.03	0.03
*Rikenellaceae RC9 gut group*	Rikenellaceae	2.25^a,b^	2.66^a,b^	1.96^b^	2.80^a^	0.31	0.04
*NK4A214 group*	Oscillospiraceae	1.02^b^	1.29^a,b^	0.98^b^	1.41^a^	0.12	0.01
*UCG-002*	Oscillospiraceae	1.11^a^	1.09^a^	0.64^b^	1.08^a^	0.40	0.02
*Schwartzia*	Selenomonadaceae	1.17	0.95	1.34	1.12	0.19	0.10
*uncultured*	Selenomonadaceae	1.20	0.72	1.42	0.72	0.37	0.10
*CAG-352*	Ruminococcaceae	0.94	0.50	0.62	0.68	0.19	0.07
*Olsenella*	Atopobiaceae	0.21^b^	0.59^a^	0.28^b^	0.27^b^	0.27	< 0.01

^1^CON, control; MOL, molasses; CWP, untreated condensed whey permeate; TCWP, treated condensed whey permeate.

^2^Effect of experimental treatment.

Similar to the solid ration the relative abundance of the genus *Succinivibrionaceae_UCG-001* which can promote propionate synthesis tended to be greater in CON and CWP (*P* = 0.06), which agrees with increased propionate concentrations in these treatments. For the genera *Treponema,* the relative abundance was greater in MOL compared to CWP (*P* = 0.02). This genus is sensitive to changes in pH (Mao et al., 2013), which concords with the decreased ruminal pH in the CWP compared to MOL observed in our companion study (Ravelo et al., 2021). The genera *Marvinbryantia,* which is classified as lignocellulose bacteria and can contribute to fibrolytic capacity of the rumen (Wei et al., 2021), had a greater relative abundance in MOL and TCWP compared to CWP (*P* = 0.03; *P* = 0.05). However, *Oribacterium* which has been identified in the rumen of cows who were fed basal forage diets (Kong et al., 2010) were decreased in MOL compared to CON (*P* = 0.04). Whether the addition of molasses to diet can be help maintain fiber degradation needs to be further explored.

The genera *Rikenellaceae RC9 gut group* has been positively correlated with ruminal pH (Qiu et al., 2022), the greatest relative abundance was observed in TCWP compared to CWP (*P* = 0.05), which corresponds to the increased pH observed previously in TCWP. Similarly, *NK4A214 group* relative abundance was increased in TCWP compared to CWP and CON (*P* = 0.04; *P* = 0.05). This genus is uncultured taxa and there role in the rumen is unclear (Amat et al., 2021). The *Oscillospiraceae UCG-002* genera has been positively correlated with NDF and ADF levels in the diet (Wei et al., 2021). The relative abundance was lower in CWP compared to all other treatments (*P* = 0.02), which can be further reflective of low conservation of fiber digestibility when incorporating CWP into the diet. Similar to the solid fraction, *Olsenella* was greater in MOL compared to all other treatments which correlates to results seen in the companion paper were lactate was present in greater concentrations in MOL. Likewise, to the solid fraction a Pearson correlation was ran on A:P ratio and pH as the differences in relative abundance in genera seem to coincide with the change observed in fermentation end products and ruminal pH.

**Figure 5.**
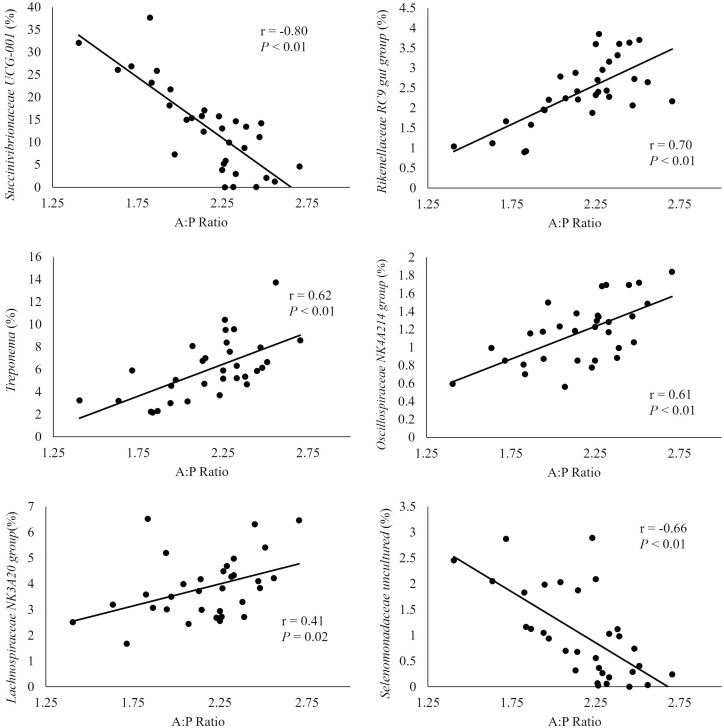
Correlation between acetate: propionate ratio (A:P ratio) in ruminal fluid and relative abundance of genera in the liquid fraction. Moderate (> 0.30) to strong (> 0.50) correlations are represented.

The correlation of the A:P ratio with genera in the liquid fraction is depicted in Figure 5, the genera *Succinivibrionaceae UCG-001* and *Selenomonadaceae uncultured* were negatively correlated with A:P ratio indicating a greater relative abundance promotes propionate over acetate production (*r* = −0.80, −0.66). The relative abundance of these bacteria was greater in CON and CWP which tended to have greater propionate molar proportion. The genera *Treponema, Lachnospiraceae NK3A20 group, Rikenellaceae RC9 gut group*, and *Oscillospiraceae NK4A214* group were positively correlated with A:P ratio (*r* = 0.62, 0.41, 0.70, 0.61) indicating that greater relative abundance promotes greater acetate molar proportions compared to propionate. For these bacteria their relative abundance was mostly increased in TCWP which tended to have greater acetate molar proportion as observed in our companion study (Ravelo et al., 2021).

**Figure 6.**
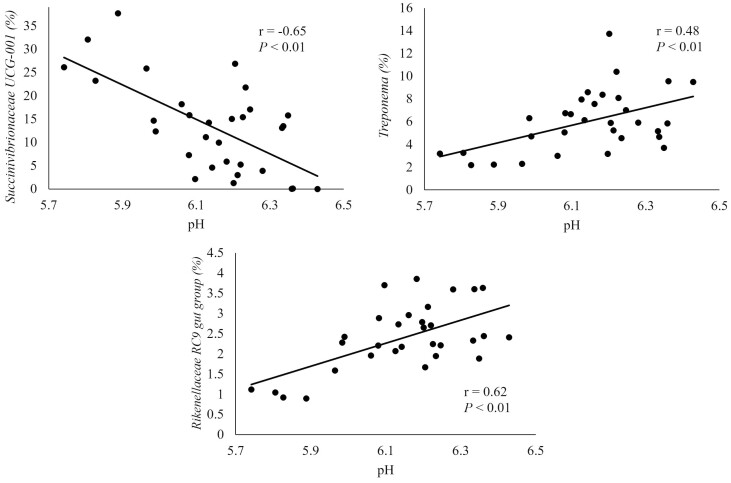
Correlation between pH in ruminal fluid and relative abundance of genera in the liquid fraction. Moderate (> 0.30) to strong (> 0.50) correlations are represented.

The correlation of pH with genera in the liquid fraction is depicted in Figure 6. The genera *Succinivibrionaceae UCG-001* was negatively correlated with pH (*r* = −0.65), thus there is a greater abundance with decreased pH. The relative abundance of this genera was greater in CON and CWP which had lower pH according to our companion study (Ravelo et al., 2021). The pH was positively correlated with *Treponema* and *Rikenellaceae RC9 gut group* (*r* = 0.48, 0.62). The relative abundance of these bacteria was increase in MOL and TCWP which had greater pH.

The incorporation of CWP for starch may have detrimental effects towards the abundance of fibrolytic bacteria. Incorporating molasses for starch has similar effects on the microbiome as CWP; however, it seems to be more conservative of fibrolytic bacteria. Additionally, molasses increases lactic acid-producing bacteria. Thus, including treated CWP in diets for dairy cows may favor relative abundance of fibrolytic bacteria and a lower abundance of lactate synthesizing bacteria.

## CONCLUSIONS

Overall, our results indicate that the replacement of starch for sugars such as molasses and CWP has an effect on the ruminal microbiome. More specifically, sugars affect bacteria that are primarily associated with synthesis of propionate and acetate and ruminal pH. To our knowledge, this is the first time that such differences are reported. These findings contribute to further the understanding of how the ruminal microbiome is related to pH, SCFA concentration, and fiber digestibility with the inclusion of more fermentable substrates in the diet.
